# Benefits of wearable-based cardiac rehabilitation interventions in secondary prevention of coronary artery disease – a systematic review and meta-analysis

**DOI:** 10.1016/j.ajpc.2025.101015

**Published:** 2025-06-06

**Authors:** Theodoros Maximidou, Ute Mons

**Affiliations:** Cardiovascular Epidemiology of Aging, Department of Cardiology, Faculty of Medicine and University Hospital Cologne, University of Cologne, Germany

**Keywords:** Coronary artery disease, Cardiac rehabilitation, Wearable devices, Activity tracker, Physical activity, Prognosis

## Abstract

**Aim:**

To assess the benefits of wearable activity trackers in the treatment and care of patients with coronary artery disease (CAD), we performed a systematic review and meta-analysis.

**Methods:**

We systematically searched databases and trial registries until March 2025 for randomized controlled trials employing wearable devices (such as activity tracker, pedometer or accelerometer) in cardiac rehabilitation (CR) interventions in patients with CAD. The outcome data were pooled using fixed-effects meta-analysis. Subgroup analyses were conducted for risk of bias, length of follow up, type of wearable and presence of additional interventions. To assess the robustness of the main findings we carried out sensitivity analyses using random effects models and exclusion of outliers. Outcomes of interest were indicators of prognosis and prognostic factors.

**Results:**

We included a total of 23 studies and synthesized data from 20 studies in meta-analyses. Meta-analysis of steps per day showed a statistically significant difference favouring the intervention (MD 1060 steps/day, 95 % CI 650 to 1460). Subgroup analyses indicated smaller effects for studies with longer follow-up periods, and for those with high risk of bias. Sensitivity analyses showed robustness of these results. Meta-analyses of rehospitalizations (RR 0.70, 95 % CI 0.52 to 0.95), 6 min walking test (MD 13.06 m, 95 % CI 0.10 to 26.03), and absolute VO_2_peak (MD 0.22 L/min, 95 % CI 0.02 to 0.49) also yielded statistically significant differences favouring the intervention. Findings from other physical performance measures favoured the intervention group without reaching significance. Anthropometric outcomes presented no consistent effect.

**Conclusions:**

Our results indicate that wearables significantly enhance effectiveness of CR by increasing physical activity, improving exercise capacity, and reducing rehospitalizations in CAD patients. This suggests that wearable-supported CR programs may positively affect prognosis in CAD. However, further research is needed to corroborate these findings and to ascertain the sustainability of these effects over the long term.

**Lay summary:**

In our study, we found that wearable activity trackers significantly increased steps per day, improved exercise capacity and reduced rehospitalizations in patients with coronary artery disease compared to usual care. These findings suggest that the implementation of wearable activity trackers into cardiac rehabilitation might improve clinical outcomes and potentially reduce the burden of the disease.

## Graphical abstract

**Figure:**
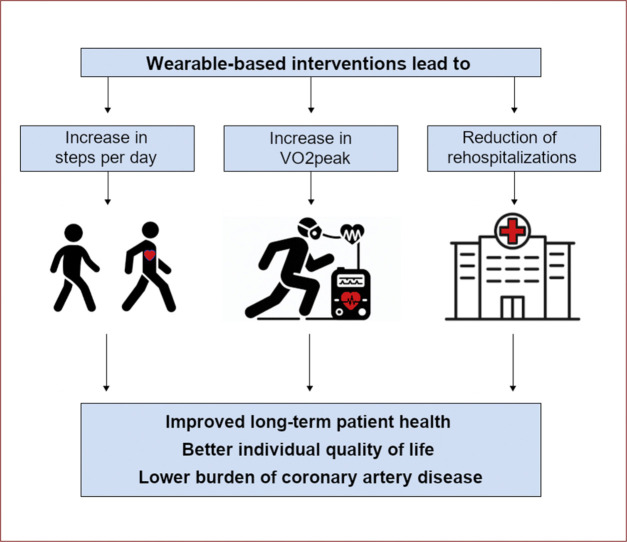

## 1 Introduction

Despite preventive strategies and treatments evolving at a fast pace throughout medicine, cardiovascular diseases, and in particular coronary artery disease (CAD), remain leading causes of morbidity and mortality worldwide. In 2006, healthcare costs attributed to CAD amounted to an estimated €105 billion in the European Union and the United Kingdom alone. When factoring in production losses and informal care, the total costs were estimated to be €169 billion [1]. These expenses are projected to increase further due to an ageing population [2].

The Global Burden of Disease Study group estimated that around 244 million people worldwide were living with CAD in 2020 and proposes that a total of 8.95 million deaths are attributable to CAD. The age-standardized prevalence and mortality of CAD has been reported to be highest in Africa, the Middle East, Eastern Europe, and Central Asia [3]. Particularly for these regions, where most of the low-to-middle income countries are located, cost-effective therapies are urgently required. Exercise-based cardiac rehabilitation (CR) plays a key role when it comes to reducing the financial burden of disease, as it has been shown to reduce cardiovascular mortality and to be cost-effective [4].

However, conventional in-person CR still requires substantial resources and personnel. A growing number of studies hence suggest that wearables improve CR effectiveness [5,6] reduce costs, and enhance patient adherence [7]. Wearable technologies comprise a variety of devices, such as smartwatches, fitness bands, waist-worn sensors, and implantable devices. They are all capable of collecting biometric data through sensors measuring variables such as heart rate, blood pressure, blood oxygen saturation, steps taken, and number of calories burned [8]. These data are collected in real time, transmitted to cloud servers, and can be accessed via software interfaces [9]. Wearable devices can also provide feedback on the collected data. For example, they may send notifications whenever the user has been sedentary for too long, and they can give positive feedback when an activity goal has been achieved [10]. In addition, gamification strategies involving game-like elements, such as rewards and leaderboards, have been used to boost physical activity (PA) levels, motivation to maintain healthy habits, and adherence to therapy [11]. Such approaches, which align with the principles of behaviorism and the theory of operant conditioning, can motivate users to increase physical activity and make healthier lifestyle choices through positive reinforcement. The effectiveness of such strategies has been demonstrated in healthy individuals [12] and is now increasingly investigated in the context of diabetes mellitus, stroke, or cardiac diseases [[13], [14], [15], [16]].

Many risk factors affecting cardiovascular health are modifiable, and evidence shows that reducing sedentary behavior, increasing physical activity, and maintaining a healthy lifestyle is effective in cardiovascular prevention [17]. An umbrella review of 39 systematic reviews highlighted that wearables can improve multiple metrics of PA, as well as body composition, blood pressure, cholesterol levels and overall fitness and wellbeing [18]. Given this research base, we hypothesize that the use of wearable devices in CR can boost physical activity and improve secondary prevention in a CAD population.

Hence, we set out to systematically review existing research and conduct a meta-analysis to determine whether the application of wearable devices in CR interventions can indeed improve prognostic factors or prognosis in persons with CAD.

## 2 Methods

We conducted a systematic review and a meta-analysis of randomized controlled trials (RCTs) to investigate the effect of wearables in CR on prognosis and prognostic factors. This study is in alignment with the Preferred Reporting Items for Systematic Reviews and Meta-Analysis (PRISMA) guidelines (see Supplementary Table 1 for checklist) [19]. The study was registered at the International Prospective Register of Systematic Reviews PROSPERO (CRD42021252651) before initiating the literature search.

### 2.1 Search strategy

We defined search terms using the PICO framework [20] and carried out database searches in May 2021 in the electronic databases PubMed and Web of Science. To include further studies published since the initial search and fetch the most up-to-date evidence, we re-ran the search in March 2023 and March 2025. We limited inclusion to studies published from 2006 onward, aligning with the widespread commercialization of wearables, which started with Nike and Apple’s collaboration in 2006 and Fitbit’s founding in 2007 [21,22]. Search terms related to the population included synonyms for CAD, like ischemic heart disease and coronary heart disease, and related terms such as cardiac, arteriosclerosis, etc. Search terms regarding the type of intervention comprised wearable, mHealth, smartwatch, fitness tracker, activity tracker and other related terms, as well as the most common brand and product names. Search terms for the outcome included terms related to disease prognosis, such as coronary events, myocardial infarction, hospitalization, or on prognostic factors, such as steps per day, body mass index (BMI), oxygen consumption (VO_2_), cardiac fitness, etc. Further, search terms specifying the study design (such as randomized and RCT) were added. The complete list of search terms is available as supplementary material in the online appendix [Supplementary Table 2]. Additionally, we searched for relevant unpublished studies in the clinical trials registers ClinicalTrials.gov and the Cochrane Central Trials Register of Controlled Trials.

### 2.2 Eligibility and inclusion criteria

To be eligible for inclusion, studies had to be RCTs assessing the use of wearables that are capable of tracking steps, acceleration or activity in patients enrolled in a CR program. Studies had to include a control group that had not been provided with any wearable device as intervention. The study population had to include participants older than 18 years of age and with a main diagnosis of CAD, myocardial infarction, acute coronary syndrome, or angina pectoris, who may have undergone coronary artery revascularization. Studies mainly including patients with heart failure as main cardiac diagnosis were not included. Studies had to include at least one outcome reflecting a prognostic factor (such as steps per day, VO_2_, cardiac fitness) or prognosis (such as cardiac infarction, rehospitalization, mortality), measured primarily through objective means such as wearables, cardiopulmonary exercise testing, or diagnostic or laboratory tests. Studies were excluded if their outcome assessments were solely based on questionnaires or self-report by patients. Additionally, eligible studies had to be published in English or German due to resource constraints on adequate translation.

### 2.3 Screening for eligibility and study selection

Identified references were imported into EndNote 20 reference manager, merged and duplicates removed. Title and abstract screening were performed to exclude references not meeting the inclusion criteria. Next, full text screening of the remaining studies was performed independently by the two authors and results were compared for accordance. Discrepancies in inclusion and exclusion decisions were resolved through discussion.

### 2.4 Risk of bias assessment

To evaluate the quality of the included studies, the two authors independently assessed the risk of bias (RoB) for each study, using the Cochrane Risk of Bias tool for randomized trials [23]. Each of the eight items in the Cochrane Risk of Bias tool was rated by low, high, or unclear risk of bias. The two authors compared their assessments and discrepancies were solved through discussion. A study’s overall RoB was rated as low if no more than one out of eight items had a high risk of bias rating, and as high, if two or more items had a high risk of bias. We used the Review Manager (RevMan) v5.4.1 to generate the plots for the risk of bias assessment [24].

### 2.5 Data extraction

We developed a standardized data extraction form based upon a template provided by the Cochrane Collaboration [25]. Data extraction was independently conducted by the two authors. The extracted results were cross-checked to minimize errors and inconsistencies.

### 2.6 Statistical analysis

Results of continuous outcomes were synthesized by performing fixed-effects meta-analyses using the inverse variance method (IV) and mean differences (MDs) served as effect measures. We had decided a priori to use the changes from baseline to the last reported timepoint when multiple timepoints were reported. Whenever reported, we used MDs and standard deviations (SDs). If MDs were not reported, we calculated the within-group MD and the between-group MD from the continuous data provided. Risk ratios (RRs) served as effect measures for binary outcomes. Here, we used fixed-effects meta-analysis along with the Mantel-Haenszel method. We performed subgroup analyses by RoB, length of follow up, type of wearables used in the intervention, and the application of additional intervention components besides wearables. All results were depicted as forest plots displaying the MDs and RRs, respectively, along with their 95 % confidence intervals (CI). To test the robustness of the results we performed sensitivity analyses by excluding potential outliers, and by re-running analyses using random-effects models. Where applicable, funnel plots were used to screen for publication bias. Statistical analyses were performed using RevMan v5.4.1 [24].

## 3 Results

**Fig. 1:**
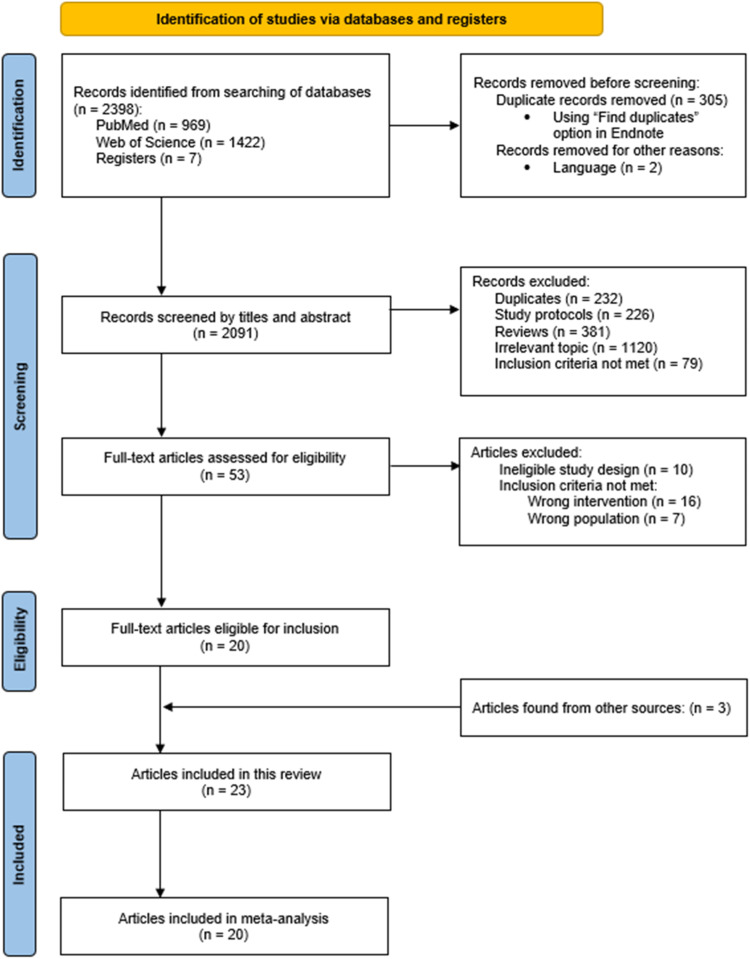
PRISMA flow diagram of study selection [19].

Database and register searches resulted in a total of 2398 references. After systematically screening titles and abstracts, 53 references remained for full text screening. After acquiring and screening the full texts, we excluded another 33 publications. By screening the reference lists, we identified three further eligible articles, ultimately resulting in 23 eligible publications (presenting results from 22 studies). The full study selection process is shown in the PRISMA flow diagram in Fig. 1.

The included studies encompassed a total of 2964 study participants. The sample sizes of individual studies ranged from 18 to 731. Five studies had been conducted in the USA, four in Australia, two in The Netherlands, two in Finland, two in China, and one each in Belgium, France, Greece, Canada, Japan, and Germany. On average, participants were 61.5 years old, with 20 % being female and 80 % male. One study included only male [26] and one study only female participants [27]. Thirteen studies provided detailed information about the study population’s history of cardiac intervention [[28], [29], [30], [31], [32], [33], [34], [35], [36], [37]]. In these studies, an average of 90 % of participants had a CAD, of whom 52 % had undergone PCI and 21 % CABG. Other primary diagnoses, such as heart failure, valvular heart diseases or intermittent claudication, were present in 10 % of the population [[28], [29], [30], [31], [32], [33], [34], [35], [36], [37]]. Four studies did not provide detailed information on participants’ disease history in relative or absolute numbers [26,[38], [39], [40]]. The summarized characteristics of the analyzed studies are shown in Table 1.

**Table 1:** Characteristics of included studies.

Author (Year)	Observation period	Population Total randomized (R) Percent male (M) Randomized to intervention group (IG) Randomized to control group (CG)	Intervention and control condition	Outcomes (unit)
**Beckie** **(2024)** [27]	3 months	R: 30 M: 0 IG: 16 CG: 14	Intervention: HerBeat coaching app + Fitbit Alta HR™ activity tracker Control: Usual care + educational material	6-minute walking distance (meters) Physical activity (questionnaire) Blood pressure (BP) (mmHg) Heart rate (HR) (beats/min) Body mass index (kg/m²) Waist circumference (WC) (cm)
**Butler** **(2009)** [28]	6 months	R: 122 M: 68 IG: 62 CG: 60	Intervention: Pedometer, step calendar for self-monitoring, physical activity information brochures, two telephone-based counselling and goal-setting sessions Control: Usual care + information brochures	Total physical activity (min/week; sessions/week) Walking (min/week; sessions/week) Metabolic equivalents at anaerobic threshold Body mass index (BMI) (kg/m²) Waist circumference (cm)
**Dodson (2025)** [41]	3 months	R: 400 M: 72.8 IG: 298 CG: 102	Intervention: Tablet-delivered mHealth program + weekly telephone coaching + Fitbit Flex 2™ activity tracker Control: Usual care	6-minute walking distance (meters) SF-12 Quality of Life (questionnaire) Angina episodes (questionnaire) ADL impairment (questionnaire)
**Duscha** **(2018)** [29]	12 weeks	R: 32 M: 81.2 IG: 21 CG: 11	Intervention: Fitbit activity tracker, tailored weekly phone-based health coaching sessions Control: Usual care	VO_2_peak (mL/kg/min; L/min) Steps per day CPX test time to exhaustion (*sec*) Low activity (min/day; min/week) Moderate-low activity (min/day; min/week) Moderate-high activity (min/day; min/week) Total active (min/day; min/week) Peak respiratory exchange ratio (peak RER)
**Frederix** **(2015)** [30]	18 weeks	R: 80 M: 82.5 IG: 40 CG: 40	Intervention: Accelerometer, weekly personalized feedback messages with step count goals Control: Usual care + blinded accelerometer for three weeks for measurement purposes	VO_2_peak (mL/kg/min; mL/min) Blood glucose (BG) (mg/dL) HbA1c ( %) Total cholesterol (CT) (mg/dL) Low-density-lipoprotein (LDL) (mg/dL) High-density-lipoprotein (HDL) (mg/dL) Triglycerides (TG) (mg/dL) Respiratory gas exchange ratio (RERmax) Heart rate at rest (beats/min) Heart rate at max (beats/min) Diastolic blood pressure at rest (mmHg) Diastolic blood pressure at max (mmHg) Systolic blood pressure at rest (mmHg) Systolic blood pressure at max (mmHg) RPP^1^rest (mmHg*beats/min) RPPmax (mmHg*beats/min) Number of rehospitalizations [1] RPP = Rate pressure product
**Furber** **(2010)** [31]	6 months	R: 222 M: 68.0 IG: 109 CG: 113	Intervention: Pedometer, step calendar for self-monitoring, physical activity information brochures, two telephone-based counselling and goal-setting sessions Control: usual care + information brochures	Total physical activity (min/week; sessions/week) Walking (min) Walking (sessions)
**Guiraud** **(2012)** [32]	8 weeks	R: 29 M: 82.8 IG: 19 CG: 10	Intervention: Accelerometer, bi-weekly telephone-based feedback and counselling sessions Control: Usual care + accelerometer for one week for measurement	Light intensity physical activity (min/week) Moderate intensity physical activity (min/week) Light active energy expenditure (kcal) Moderate active energy expenditure (kcal) Total active energy expenditure (kcal)
**Hakala** **(2021)** [38]	12 months	R: 59 M: 81 IG: 29 CG: 30	Intervention: Fitbit activity tracker, two face-to-face support sessions, monthly prompts to engage in physical activity, monthly feedback by physiotherapist Control: Usual care	Changes in light physical activity Changes in moderate-to-vigorous PA Changes in steps per day
**Houle** **(2011)** [33]	12 months	R: 65 M: 78.5 IG: 32 CG: 33	Intervention: Pedometer, diary for self-monitoring, seven telephone-based and face-to-face support sessions with clinical nurse specialist Control: Usual care	Change in average daily steps LDL (mmol/L) Apolipoprotein-B (g/L) HDL (mmol/L) Triglycerides (TG) (mmol/L) CT/HDL ratio Fasting blood glucose (mmol/L) Waist circumference (cm) Systolic blood pressure (mmHg) Diastolic blood pressure (mmHg) Heart rate (beats/min)
**Houle** **(2012)** [42]	12 months	R: 65 M: 78.5 IG: 32 CG: 33	Intervention: Pedometer, diary for self-monitoring, seven telephone-based and face-to-face support sessions with clinical nurse specialist Control: Usual care	Average daily steps % with > 7500 average daily steps % achieving LDL ≤ 2 mmol/L % achieving CT/HDL ≤ 4 % achieving WC treatment target (men: <102 cm, women: <88 cm) % achieving TG < 1.7 mmol/L % achieving HDL treatment target (men < 1.0mmol/L, women <1.3mmol/L) % achieving blood pressure < 130/85 mmHg % achieving fasting BG < 6.1 mmol/L
**Indraratna** **(2022)** [36]	6 months	R: 164 M: 79.3 IG: 81 CG: 83	Intervention: Activity tracker, tele-clinical care smartphone app, sphygmomanometer, weighing scale, three weekly health promotion push notifications Control: Usual care	Incidence of 30-day hospital readmissions Total hospital readmissions Mortality Nonfatal myocardial infarction Nonfatal stroke Major adverse cardiovascular events Systolic blood pressure (mmHg) Weight (kg) Waist circumference (cm) 6-minute walking distance (meters) LDL (mmol/L)
**Izawa** **(2012)** [39]	4 weeks	R: 126 M: 80.6 IG: 63 CG: 63	Intervention: Accelerometer, exercise calendar for self-monitoring Control: Usual care + Accelerometer for two weeks for measurement	Average daily number of steps Average daily energy expenditure (kcal)
**Kaminsky** **(2013)** [34]	8 weeks	R: 18 M: 77.8 IG: 10 CG: 8	Intervention: Pedometer + individualized step count goals Control: Usual care	Steps per day Moderate-to-vigorous physical activity (min/d)
**Lahtio** **(2023)** [43]	12 months	R: 59 M: 81 IG: 29 CG: 30	Intervention: mCoach smartphone + Fitbit Charge 2™ activity tracker Control: Usual care	6-minute walking distance (meters) Waist circumference (cm) Body mass index (kg/m²) Quality of Life (questionnaire)
**Mitropoulos** **(2024)** [44]	24 weeks	R: 30 M: 73 IG: 15 CG: 15	Intervention: Home-based exercise program + Polar OH1™ activity tracker Control: Usual care	VO₂peak (mL/kg/min) Predicted VO2peak ( %) Exercise time (min) Steps per day Mean daily distance (km) Mean daily calories (kcal) Lean muscle mass (kg) Fat mass ( %)
**Ozemek** **(2020)** [40]	12 weeks	R: 50 M: 76.0 IG: 21 CG: 21	Intervention: pedometer + step count goals Control: Usual care	Average change in steps per day Sedentary time (min/day) Light physical activity (min/day) Moderate-to-vigorous physical activity (min/d)
**Patterson** **(2023)** [45]	12 months	R: 120 M: 77.5 IG: 60 CG: 60	Intervention: ToDo-CR smartphone app + ActivPAL™ activity tracker (pocket-worn) Control: Usual care	Hospitalizations Sedentary time (min/day) Body mass index (kg/m²) Waist circumference (cm) Quality of life (questionnaire) Percentage of SB/d (SB/wear time) Average duration of SB bouts (min) Number of SB bouts/d Number of SB breaks/d Moderate-to-vigorous physical activity (min/d) LPA (min/d) Steps per day
**Su** **(2021)** [46]	12 weeks	R: 146 M: 83.6 IG: 73 CG: 73	Intervention: Nurse-led eHealth CR program (WeChat platform) + Mi Band 2™ activity tracker Control: Usual care (pedometer for data collection)	Steps per day Sitting time (questionnaire) HPLP-II-Score (questionnaire) Smoking cessation (yes/no) Cardiac self-efficacy (questionnaire) Health-related quality of life (questionnaire) Psychological distress (questionnaire) Blood pressure (mmHg) Body mass index (kg/m²) Hospital admissions Emergency visits
**Su** **(2024)** [47]	12 weeks	R: 43 M: 84 IG: 21 CG: 22	Intervention: Cardiac Telerehabilitation (CTR) system + Mi Band 2™ activity tracker Control: Usual care (pedometer for data collection)	Steps per day Health-promoting lifestyle (questionnaire) Feasibility and usage metrics
**Ter Hoeve** **(2018)** [35]	18 months	R: 731 M: 82.0 IG1: 246 IG2: 240 CG: 245	Intervention: Pedometer, three face-to-face physical activity group counselling sessions, three general lifestyle face-to-face group counselling sessions Control: Usual care	Moderate-to-vigorous physical activity ( % of wear time) Step count (steps per minute of wear time) Sedentary behavior ( % of wear time) Prolonged moderate-to-vigorous physical activity >10 mins ( % of wear time) Prolonged SB ( % of wear time) Achieving step guidelines (6500 steps per day; %) Achieving moderate-to-vigorous physical activity guidelines (150 min/week; %)
**Treskes** **(2020)** [37]	6 months	R: 200 M: 78.0 IG: 100 CG: 100	Intervention: Withings activity tracker, blood pressure monitor, weight scale, single-lead ECG device Control: Usual care	Number of patients with controlled blood pressure (< 139/89 mmHg) Mortality Hospitalizations (nonfatal cardiac adverse events)
**Van Bakel** **(2023)** [48]	12 weeks	R: 212 M: 77 IG: 108 CG: 104	Intervention: eCoaching + App + ActivPAL™ activity tracker (pocket-worn) Control: Usual care	Device-measured sedentary time (min/day) Physical activity (questionnaire) Quality of life (questionnaire) Cardiovascular risk score
**Vogel** **(2017)** [26]	12 weeks	R: 36 M: 100 IG: 19 CG: 17	Intervention: Polar loop activity tracker Control: Usual care	Mean maximum power (watt)

### 3.1 Intervention characteristics

Out of the twenty two studies, fourteen used wrist-worn activity trackers to provide feedback on daily activity [26,27,32,[36], [37], [38],^41^,[43], [44], [45], [46], [47], [48]] two used accelerometers [30,39] and seven studies used pedometer feedback [28,31,[33], [34], [35],^40^]. A total of nine studies used special smartphone apps as additional interventions to provide motivational gamification progress tracking and to acquire data and deliver feedback for the therapists [29,36,37,41,43,[45], [46], [47], [48]]. Four studies offered additional phone consultations for the intervention arm [28,32,33,41] three studies offered live coachings [27,44,47] and one study delivered phone calls and in-person meetings for the intervention group (IG) [42]. Further additional intervention components included step calendars for self-monitoring [28,31,33,39] information brochures [28] wearables other than activity trackers, accelerometers or pedometers [36,37] educational push notifications [36] and questionnaires [28,31]. The mean duration of intervention was 25 weeks, ranging from 26 days to 18 months, with a median follow-up period of 18 weeks.

### 3.2 Risk of bias analysis

Regarding risk of bias, ten studies had a low overall RoB [33,[37], [38], [39], [40], [41],^43^,^44^,^46^,^48^] while twelve had a high overall RoB [[26], [27], [28], [29], [30], [31], [32],[34], [35], [36],^42^,^45^]. Due to the impossibility of blinding of participants and personnel, all studies had a high risk of performance bias. Risk of selection bias was unclear for most studies, due to insufficient reporting of allocation concealments. However, nine studies did report concealed allocation strategies and were rated with low risk of selection bias [27,31,41,[43], [44], [45], [46], [47], [48]]. Four studies failed to report whether the outcome assessment was blinded and were rated with unknown risk of detection bias [26,28,31,35,43] while all other studies assessed outcomes through blinded activity trackers [[28], [29], [30],[32], [33], [34],[36], [37], [38], [39], [40],^42^]. Three studies had a high risk for attrition bias regarding all outcomes [28,35,27] while one was rated high risk for anthropometric measures and low for assessment of rehospitalization [36]. Four studies presented an unclear risk for attrition [29,31,40,47]. Furthermore, ten studies were rated with a high risk of reporting bias due to the lack of a specified primary outcome, lack of a pre-registered study protocol or inconsistencies between registered study protocol and final selection of results in the publication [26,27,[29], [30], [31], [32],^34^,^35^,^42^,^45^]. The summarized results of the RoB-analysis are presented in Table 2.

**Table 2:** Risk of bias assessment (RSG = random sequence generation, AC = allocation concealment [1], anthropometric measures [2], hospital readmissions, = low RoB, = high RoB, = unclear RoB).

### 3.3 Outcomes

#### 3.3.1 Physical activity parameters

Eleven studies investigated the effects of wearables on participants’ steps per day [29,33,34,[38], [39], [40],[44], [45], [46], [47], [48]]. Six studies examined the amount of moderate-to-vigorous physical activity (MVPA) [29,32,34,38,40,45] with four reporting the results as minutes per day [29,34,40,45] and two as minutes per week [32,38]. Three studies assessed the amount of light physical activity (LPA) in minutes per day [29,38,40] and four in minutes per week. While three studies measured the total physical activity (TPA) in minutes per week [28,29,31]. Two studies examined the number of minutes per week spent walking [28,31]. One study assessed the mean daily distance taken and mean daily activity [44].

**Fig. 2:**
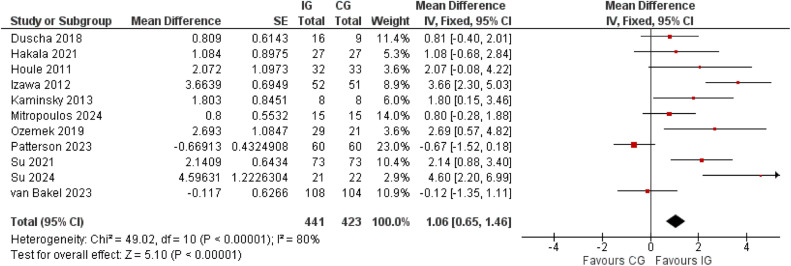
Meta-analysis of the mean changes in steps per day; reported in increments of 1000 steps.

**Fig. 3:**
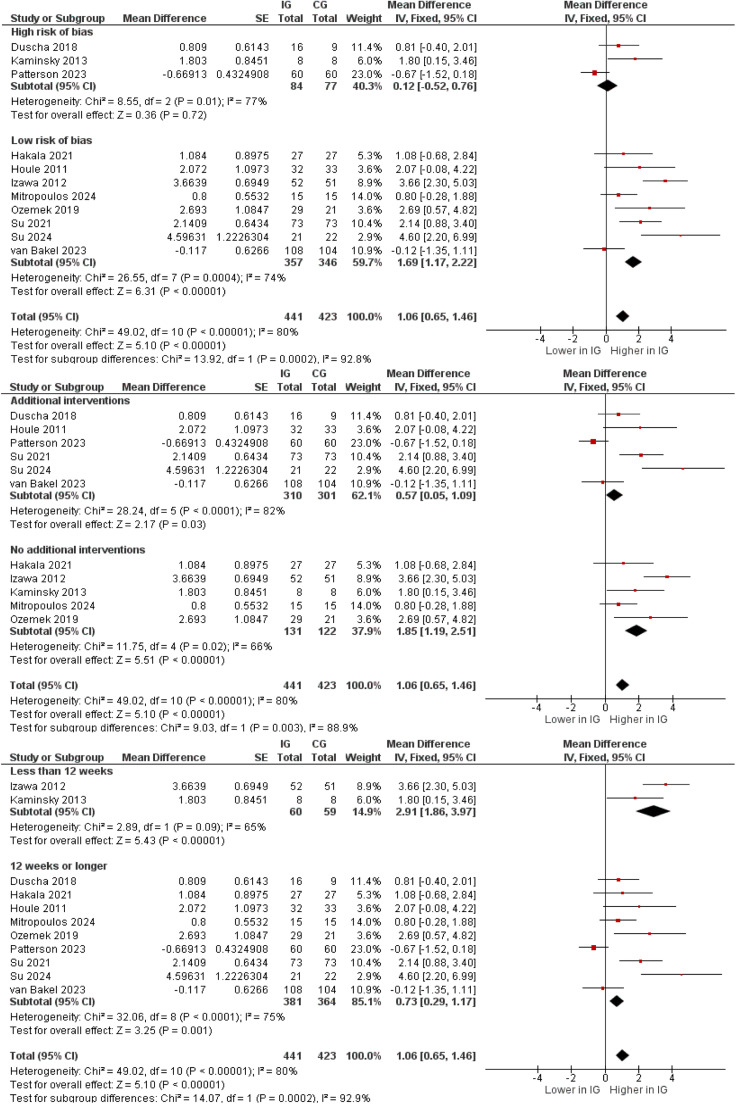
Subgroup analyses of the mean changes in steps per day (reported in increments of 1000 steps) by use of additional intervention components, by duration of intervention, and by risk of bias.

Regarding steps per day, a statistically significant mean difference favoring the intervention was found (MD 1.06, 95 % CI 0.65 to 1.46), translating to a between group difference of 1060 steps per day. [Fig. 2] Sensitivity analyses showed similar summary estimates when outliers were excluded [Supplementary figure 23] or when compared to random effects meta-analysis [Supplementary figure 24]. In subgroup analysis, the summary estimate was significantly lower in studies with additional intervention components compared to those without (MD 0.57, 95 % CI 0.05 to 1.09 vs. MD 1.85, 95 % CI 1.19 to 2.51; Fig. 3). Sensitivity analyses however suggested no significant difference between these subgroups after exclusion of outliers (MD 1.03, 95 % CI 0.36 to 1.71 vs. MD 0.12, CI −0.52 to 0.76; details not shown). Subgroup analysis for RoB revealed a significantly higher MD in steps per day for the studies with low RoB compared to high RoB (MD 1.69, 95 % CI 1.17 to 2.22 vs. MD 0.12, 95 % CI −0.52 to 0.76; Fig. 3). Here as well, removal of the outlier studies led to a non-statistically significant difference of summary estimates (details not shown). Subgroup analysis by the length of follow up showed that studies with <12 weeks of follow up measured a significantly higher MD than those with a minimum of 12 weeks of follow up (MD 2.91, 95 % CI 1.86 to 3.97 vs. MD 0.73, 95 % CI 0.29 to 1.17). [Fig. 3] The use of pedometers for intervention instead of activity trackers or accelerometers resulted in significantly higher intervention effects (MD 2.12, 95 % CI 1.00 to 3.24 vs. MD 0.89, 95 % CI 0.46 to 1.33) [Supplementary figure 25]. Yet again after exclusion of outlier studies the difference did not reach statistical significance (MD 0.91, 95 % CI 0.34 to 1.47 vs. MD 2.12, 95 % CI 1.00 to 3.24; details not shown).

Meta-analysis by MVPA, and TPA showed results favoring the IG, with results for MVPA and TPA showing significantly higher activity in minutes per week in the IG (MVPA: MD 62.10, 95 % CI 31.06 to 93.13; TPA: MD 86.81, 95 % CI 32.47 to 141.16). For LPA in minutes per week however, no benefits for the intervention group were observed (MD 60.67, 95 % CI −37.72 to 159.05). Walking minutes per week were found to be significantly higher in the IG (MD 72.95, 95 % CI 33.29 to 112.61). [Supplementary figures 1–4]

A funnel plot of the pooled studies for the meta-analysis of steps per day showed a slightly asymmetrical distribution, indicating a probable publication bias. On the one hand there is a lack of small studies with negative or null results, while on the other hand large studies with statistically significant effects favoring the IG are missing as well. [Supplementary figure 26]

#### 3.3.2 Physical performance measures (PPM)

Four studies assessed the difference in the 6-minute walking test (6MWT) [27,36,41,43]. Three studies conducted measurement of the peak oxygen consumption (VO_2_peak), two in absolute (L/min) [29,30] and three in relative rates (mL/kg/min) [29,30,44]. One study assessed the peak respiratory exchange ratio (RERpeak) [29] and one the maximum respiratory exchange ratio (RERmax) [30]. One study reported the metabolic equivalent of task at the anaerobic threshold (METs AT) [28] another one the mean maximum power (MMP) [26] and yet another study values of a maximal treadmill cardiopulmonary exercise test (CPX) [29].

Meta-analysis by 6MWT yielded a statistically significant effect favoring the IG (MD 13.06 m, 95 % CI 0.10 to 26.03). [Supplementary figure 22] For absolute VO_2_peak in L/min also a significant difference favoring the IG was seen (MD 0.28, 95 % CI 0.06 to 0.49). For the relative (weight adapted) VO_2_peak in mL/kg/min the effect did not reach statistical significance (MD 0.83, 95 % CI −1.34, 3.00). [Supplementary figures 5 and 6]

As a post-hoc analysis, to combine the different PPM, we performed a random effects meta-analysis using standardized mean difference calculation. Because the same two studies measured both absolute and relative VO_2_peak, as well as the RERpeak and RERmax [29,30] we compared each of them separately with the METs AT and mean maximum power. All these post-hoc analyses showed statistically significant effects favoring the intervention group. Pooling of absolute VO2peak, METs AT, MMP, and 6MWT presented the greatest effect in favor of the intervention (SMD 0.25, 95 % CI 0.07 to 0.44). [Supplementary figures 7–10]

#### 3.3.3 Rehospitalizations and cardiovascular events

**Fig. 4:**
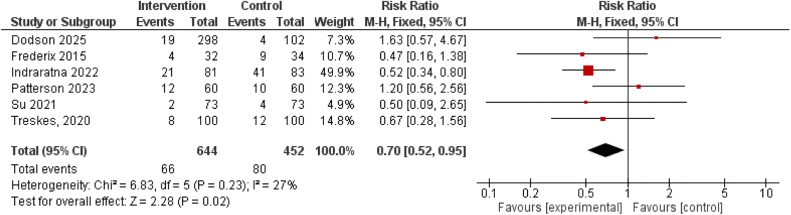
Meta-analysis of the rehospitalization rates.

Six studies reported rehospitalizations during follow up, and meta-analysis yielded a significantly lower rate of rehospitalizations in the IG (RR 0.70, 95 % CI 0.52 to 0.95). [Fig. 4] [30,36,37,41,45,46] Two studies further reported fatal and non-fatal cardiovascular events. For both outcomes, lower rates were observed in the IG, but the pooled summary estimates did not reach statistical significance (Fatal: RR 0.51, 95 % CI 0.13 to 2.00; non-fatal: RR 0.76 95 % CI 0.34 to 1.70). [Supplementary figures 11–12] [36,37]

#### 3.3.4 Blood parameters and anthropometric measures

Six studies assessed waist circumference (WC) [27,28,33,36,43,45] with one of them assessing it separately for women and men [28] while another examined only women [27]. Two studies measured the MD for blood glucose (BG), triglycerides (TG), and high-density lipoprotein (HDL) [29,30] while three studies analyzed concentrations of low-density lipoprotein (LDL) [29,30,36]. Five studies reported changes in BMI [45]. Two studies reported changes in heart rate (HR) [30,33] four in diastolic blood pressure (DBP), [27,30,33,46] and five studies assessed systolic blood pressure (SBP) [27,30,33,36,46].

Meta-analysis for WC yielded a statistically significant difference favoring the IG, while both groups showed an overall increase in WC (MD −1.08, 95 % CI −2.14 to −0.03). For the other outcomes, no statistically significant differences between IG and CG were seen. [Supplementary figures 13–19].

#### 3.3.5 Other outcomes

Several additional outcomes were reported but could not be pooled in a meta-analysis. These include age predicted peak heart rate, distance in miles per day/week, floors per day/week [29] HbA1c, total cholesterol (CT), maximum heart rate (HRmax), rate pressure product at rest (RPPrest) and at maximum capacity (RPPmax) [30] ApoB, total cholesterol to HDL-ratio (TC/HDL), smoking status [33] sedentary time in minutes per day and in percent daily time, LPA and MVPA in percent daily time [40] sedentary behavior (SB) in percent of wear time, prolonged SB and prolonged MVPA in percent of wear time, achieving step guidelines, achieving MVPA guidelines [35].

One study found that HbA1c increased significantly in the IG, while no change was observed in the CG, but the between-group analysis did not reach significance (*p* = 0.063) [30]. BMI showed no significant between group differences [28]. HRmax increased significantly both in the IG and the CG, with no significant difference in the between-group analysis (*p* = 0.311) [30]. RPPmax increased significantly in the IG without significant between-group differences while RPPrest showed non-significant results [30]. For both floors climbed per day and per week, a significant increase in the IG was reported, and a non-significant reduction of floors climbed in the CG, without a significant between-group difference [29]. Changes in age predicted peak heart rate, and miles per day/week were nonsignificant between groups [29]. Results for SB and prolonged SB in percent of wear time, and achieving MVPA guidelines showed no significant between-group differences, while prolonged MVPA in percent of wear time, and achieving step guidelines showed results favoring the IG (prolonged MVPA: *p* = 0.028; step guidelines: *p* = 0.033) [35]. Sedentary time in minutes per day and in percent daily time, as well as LPA and MVPA in percent daily time did not show significant differences [40]. Also, no significant between-group differences were found for TC, ApoB in g/L, TC/HDL, and smoking status [33].

Two publications reported the percentage of participants that achieved predefined treatment targets for steps per day, physical activity, LDL, CT/HDL, WC, TG, HDL, arterial blood pressure, or BG [35,42]. Out of these outcomes only the percentage of patients who achieved steps per day guidelines was significantly higher in the IG than in the CG. These results could not be pooled in meta-analysis though, as both studies had set different treatment targets. Assessments through questionnaires were made by several studies but were not analyzed in this review.

## 4 Discussion

This systematic review and meta-analysis suggest several beneficial prognostic effects of wearables when used as part of interventions to promote physical activity in patients with CAD, particularly on physical activity level and on risk of rehospitalization.

The most significant finding was observed for the number of steps per day. Eleven studies could be pooled in a meta-analysis, which yielded a mean difference of 1060 steps per day in favor of the IG. The results on PPM were somewhat inconsistent. Absolute VO_2_ was found to be significantly higher in the IG. Relative VO_2_, on the other hand, also tended to be higher in the IG, but the mean difference did not reach significance. Post-hoc analysis combining the different PPM could confirm these findings, by yielding statistically significant differences in favor of the IG. Generally, although not all PA-related outcomes showed significant improvements in meta-analysis, a clear trend towards improvement was observed. The small number of studies, the mostly small sample sizes, and the high diversity in tests measuring PPM might have contributed to the inconsistencies in results and the lack of statistical significance. Hence, larger studies should be conducted, especially as wearable technologies are evolving at a fast pace.

For steps per day, we observed a substantial increase in daily step counts for patients using wearables, with an average increase from 5017 to 9566, an increment of 4549 steps. When considering the latest research on daily step goals for healthy individuals, this increment of daily steps could be associated with significant reductions in all-cause and cause-specific mortality. A recent meta-analysis of general population cohort studies revealed that the increment of steps per day by 1000 was associated with a significant reduction in all-cause mortality by 15 %. Furthermore, an increment by 500 steps per day was associated with a significant reduction of cardiovascular mortality by 7 % [49]. This is also supported by Stens et al. who found an optimal dose of 8763 steps per day for risk reduction of all-cause mortality and incident CVD [50] which is in a similar range as the average daily post-intervention step count found in our study (9566). While there are no specific guidelines for step count targets for secondary prevention in CAD or cardiovascular disease in general, evidence suggests that a daily step count of 6500 to 8500 might prevent disease progression or even induce regression [51]. Hence, the results of our meta-analysis, which revealed an average increase of 4549 steps per day over the follow up period in the intervention group, and a benefit of 1060 steps above and beyond the control condition, hold clear clinical relevance. Bringing together these findings, it becomes apparent that implementing wearables in CR could potentially reduce both all-cause and cardiovascular mortality in the long-term.

The results of the meta-analysis of rehospitalizations also support this assumption. The rate of rehospitalizations was significantly lower in the IG compared to usual care for a pooled collective of 1096 patients. It is known that rehospitalizations in patients with CAD are associated with increased mortality and a higher risk for interventions [52]. Even the economic burden of disease may likely be affected since readmissions have been associated with a significant increase in healthcare costs [53,54]. However, further studies are needed to corroborate our findings.

Our results are also in line with those from similar studies. Another systematic review and meta-analysis synthesized data from ten RCTs studying the impact of wearable activity trackers in CR on step counts and aerobic capacity. The study found that use of activity trackers significantly increased both daily step counts and aerobic capacity among CR participants [55]. Moreover, a recent meta-analysis focused on the effect of wearable based interventions with feedback in a population of patients with cardiovascular disease. They found these interventions to significantly increase the number of daily steps, as well as the values of the 6-minute walking test. VO_2_peak was found to be higher in the IG, but the difference was not statistically significant [56]. To our knowledge, our study is the most up to date systematic review and meta-analysis, uniquely presenting a broad range of outcomes in a specific CAD-patient collective. In contrast to previous reviews, our study’s explicit focus on CAD makes it more applicable for clinical implementation in this specific patient group. Furthermore, our analysis covered multiple domains of outcomes and is the first meta-analysis to assess both physical activity outcomes and hard endpoints such as rehospitalization rates, offering a broader perspective on secondary prevention in CAD patients.

While no clear trends were seen for blood parameters and anthropometric measures, it is important to recognize that these measures are affected by numerous confounders, including diet, alcohol consumption, or tobacco use. They might therefore not be the most suitable surrogate markers for assessing the effectiveness of wearables. These markers have also only been investigated in a small number of studies, so that no conclusive inferences can be made.

Some studies included in our analysis employed additional intervention components besides wearables, which might also affect the results and their comparability. These additional components consisted of motivational or supportive phone calls [28,29,31,32] automated messages [29,30,36,38] and face-to-face consultations [33,35]. Interestingly, subgroup-analysis for steps per day showed a significant difference between studies with and without additional interventions, pointing towards no added value of such additional intervention elements. While this suggests that simpler interventions might be more effective than more complex multi-component interventions, the diversity of the additional intervention elements makes this result difficult to interpret. Generally, it would be expected that the delivery of motivational messages or push notifications indeed rather enhances the effect of wearables, since the latest developments of these technologies have made them interactive and capable of delivering automated feedback [57]. Gamification elements, such as social leaderboards or peer-group comparison of step counts, could also potentially further enhance the effectiveness of wearables. As the studies included in this review mostly used older generation wearables that lack these functions, future studies should evaluate newer generation wearables and integration of gamification elements for further validation in clinical settings.

The follow up periods of the included studies ranged from 1 to 18 months with an average of roughly 6 months. To determine whether the observed benefits are maintained beyond CR and impact cardiovascular prognosis also in the long term, future studies should have longer follow-up periods, ideally over several years. Considering the results of this study and of similar studies on wearables, it seems plausible that a standardized use of wearables in CR could have a positive and long-term impact on the individual outcome of CAD, even beyond CR.

Importantly, none of the screened studies reported any kind of adverse events directly related or attributable to the wearable use. This is also reported in a review on wearables by Ashur et al [55]. It is thus fair to say that wearables are a safe-to-use intervention. Moreover, studies evaluating the impact on health care costs emphasize that wearables could lead to a significant cost reduction [[58], [59], [60]]. A McKinsey report published in 2016 indicates that a shift from in-person visits to tele-consultations would lead to a cost reduction in healthcare spending by $25 billion to $40 billion in the USA [61]. And cost-effectiveness might even increase, considering that production costs as well as prices might decrease further in the future, with more and more companies entering the market.

When interpreting the findings of this study, several limitations should be considered. As the studies included in this review revealed a high diversity in outcomes examined, many parameters were only assessed by few studies, which reduces the confidence in these estimates. Also, the diversity in intervention design and elements as well as in the types of wearables limits the comparability and generalizability of the findings of the meta-analyses. Another limitation is related to the quality of the studies analyzed, as twelve of the 22 included studies had a high overall RoB rating. Of these, ten studies had not clarified whether their outcomes were pre-specified, which particularly introduces a high risk of reporting bias, possibly leading to an overestimation of effect estimates. All the studies had a high risk of performance bias, which is due to the nature of the intervention, where blinding of participants is difficult, possibly leading to an overestimation of effect estimates. Generally, because wearable devices are still just scarcely adopted in mainstream healthcare, the number of studies conducted in this field is still small, and the included studies had limited sample sizes. Finally, the title and abstract screening was conducted by only one researcher, increasing the risk of missing eligible studies. However, to mitigate this risk, the title and abstract screening was conducted in a conservative manner and full text screening was conducted by two researchers independently. Data extraction was also done by two researchers independently to limit the risk of errors. A strength of this study is that the study protocol was pre-registered on Prospero. It should be highlighted as well that – to the best of our knowledge – this is the first systematic review to assess the effect of wearables on CAD prognosis and prognostic factors.

## 5 Conclusions

Our findings indicate that wearables significantly enhance the effectiveness of CR in increasing physical activity, physical performance, and reducing rehospitalizations in patients with CAD. However, further large-scale trials are warranted to support these findings and to develop concrete step count recommendations for these patients. Therefore, it is recommended to conduct international multi-center RCTs with large sample sizes and extended follow-up periods to address heterogeneity and test the findings of our meta-analysis.

This study aligns with earlier research in other types of cardiovascular patients, indicating a consistent pattern: wearable activity trackers seem to boost physical activity level and lower the risk of adverse events, such as rehospitalizations. Considering that a significant increment of daily activity lowers the risk for cardiovascular and all-cause mortality, it is reasonable to infer that wearables could provide a benefit for secondary prevention and the prognosis of patients with CAD. Nevertheless, these findings require further validation, particularly regarding their long-term effects.

## Funding

UM is supported by Marga and Walter Boll Foundation, Kerpen, Germany. The funder had no role in study design; collection, management, analysis and interpretation of data; writing of the manuscript or the decision to submit it for publication.

## CRediT authorship contribution statement

**Theodoros Maximidou:** Writing – original draft, Visualization, Resources, Project administration, Methodology, Investigation, Formal analysis, Data curation, Conceptualization. **Ute Mons:** Writing – review & editing, Supervision, Project administration, Methodology, Data curation.

## Declaration of competing interest

The authors declare the following financial interests/personal relationships which may be considered as potential competing interests:

Ute Mons reports financial support was provided by Marga and Walter Boll Foundation. If there are other authors, they declare that they have no known competing financial interests or personal relationships that could have appeared to influence the work reported in this paper.

## Appendix Supplementary materials

Image, application 1
