# Supplementary material for: Benefits of wearable-based cardiac rehabilitation interventions in secondary prevention of coronary artery disease – a systematic review and meta-analysis
Source: Am J Prev Cardiol. 2025 Jun 6;23:101015. doi: 10.1016/j.ajpc.2025.101015 (PMC12206010; doi:10.1016/j.ajpc.2025.101015)

**Benefits of wearable-based cardiac rehabilitation interventions in secondary prevention of coronary artery disease – a systematic review and meta-analysis**

**Supplementary material**

Index P. 1

Supplementary table 1 – Prisma checklist P. 2 - 3

Supplementary table 2 – Search terms P. 4

Supplementary figure 1 – MVPA (min per week) P. 5

Supplementary figure 2 – LPA (min per week) P. 5

Supplementary figure 3 – TPA (min per week) P. 5

Supplementary figure 4 – Walking (min per week) P. 5

Supplementary figure 5 – relative VO2peak [mL/kg/min] P. 6

Supplementary figure 6 – absolute VO2peak [L/min] P. 6

Supplementary figure 7 – PPM by absolute VO2peak, METs at AT, MMP, and 6MWT P. 6

Supplementary figure 8 – PPM by relative VO2peak, METs at AT, MMP, and 6MWT P. 7 Supplementary figure 9 – PPM by METs at AT, MMP, Peak RER, RERmax, and 6MWT P. 8

Supplementary figure 10 – PPM by CPX, METs at AT, MMP, relative VO2peak, 6MWT P. 9

Supplementary figure 11 – Fatal cardiovascular events P. 9

Supplementary figure 12 – Non-fatal cardiovascular events P. 10

Supplementary figure 13 – Waist circumference (mg/dL) P. 10

Supplementary figure 14 – LDL (mg/dL) P. 10

Supplementary figure 15 – HDL (mg/dL) P. 11

Supplementary figure 16 – Triglycerides (mg/dL) P. 11

Supplementary figure 17 – Glucose (mg/dL) P. 11 Supplementary figure 18 – Systolic blood pressure (mmHg) P. 11

Supplementary figure 19 – Diastolic blood pressure (mmHg) P. 12

Supplementary figure 20 – Sedentary time (min) P. 12

Supplementary figure 21 – BMI (kg/m²) P. 12

Supplementary figure 22 – 6-minute walking test (m) P. 12

Supplementary figure 23 – Sensitivity analysis of steps per day without outlier studies P. 13

Supplementary figure 24 – Sensitivity analysis of steps per day with random-effect model P. 13

Supplementary figure 25 – Subgroup analysis of steps per day by type of wearable P. 14

Supplementary figure 26 – Funnel plot of steps per day P. 14

**Supplementary table 1 – Prisma checklist**

| **Section and Topic** | **Item #** | **Checklist item** | **Location where item is reported** |
| --- | --- | --- | --- |
| **TITLE** | | |  |
| Title | 1 | Identify the report as a systematic review. | P. 1 |
| **ABSTRACT** | | |  |
| Abstract | 2 | See the PRISMA 2020 for Abstracts checklist. | P. 1 |
| **INTRODUCTION** | | |  |
| Rationale | 3 | Describe the rationale for the review in the context of existing knowledge. | P. 1 |
| Objectives | 4 | Provide an explicit statement of the objective(s) or question(s) the review addresses. | P. 1 |
| **METHODS** | | |  |
| Eligibility criteria | 5 | Specify the inclusion and exclusion criteria for the review and how studies were grouped for the syntheses. | P. 3 |
| Information sources | 6 | Specify all databases, registers, websites, organisations, reference lists and other sources searched or consulted to identify studies. Specify the date when each source was last searched or consulted. | P. 2 |
| Search strategy | 7 | Present the full search strategies for all databases, registers and websites, including any filters and limits used. | Supplementary Table 1 |
| Selection process | 8 | Specify the methods used to decide whether a study met the inclusion criteria of the review, including how many reviewers screened each record and each report retrieved, whether they worked independently, and if applicable, details of automation tools used in the process. | P. 3 |
| Data collection process | 9 | Specify the methods used to collect data from reports, including how many reviewers collected data from each report, whether they worked independently, any processes for obtaining or confirming data from study investigators, and if applicable, details of automation tools used in the process. | P. 3 |
| Data items | 10a | List and define all outcomes for which data were sought. Specify whether all results that were compatible with each outcome domain in each study were sought (e.g. for all measures, time points, analyses), and if not, the methods used to decide which results to collect. | Supplementary Table 1 |
|  | 10b | List and define all other variables for which data were sought (e.g. participant and intervention characteristics, funding sources). Describe any assumptions made about any missing or unclear information. | Supplementary Table 1 |
| Study risk of bias assessment | 11 | Specify the methods used to assess risk of bias in the included studies, including details of the tool(s) used, how many reviewers assessed each study and whether they worked independently, and if applicable, details of automation tools used in the process. | P. 3 |
| Effect measures | 12 | Specify for each outcome the effect measure(s) (e.g. risk ratio, mean difference) used in the synthesis or presentation of results. | P. 3 |
| Synthesis methods | 13a | Describe the processes used to decide which studies were eligible for each synthesis (e.g. tabulating the study intervention characteristics and comparing against the planned groups for each synthesis (item #5)). | N / A |
|  | 13b | Describe any methods required to prepare the data for presentation or synthesis, such as handling of missing summary statistics, or data conversions. | P. 5 |
|  | 13c | Describe any methods used to tabulate or visually display results of individual studies and syntheses. | P. 3 |
|  | 13d | Describe any methods used to synthesize results and provide a rationale for the choice(s). If meta-analysis was performed, describe the model(s), method(s) to identify the presence and extent of statistical heterogeneity, and software package(s) used. | P. 5 |
|  | 13e | Describe any methods used to explore possible causes of heterogeneity among study results (e.g. subgroup analysis, meta-regression). | P. 3 |
|  | 13f | Describe any sensitivity analyses conducted to assess robustness of the synthesized results. | N / A |
| Reporting bias assessment | 14 | Describe any methods used to assess risk of bias due to missing results in a synthesis (arising from reporting biases). | P. 3 |
| Certainty assessment | 15 | Describe any methods used to assess certainty (or confidence) in the body of evidence for an outcome. | N / A |
| **RESULTS** | | |  |
| Study selection | 16a | Describe the results of the search and selection process, from the number of records identified in the search to the number of studies included in the review, ideally using a flow diagram. | P. 3 + 4 |
|  | 16b | Cite studies that might appear to meet the inclusion criteria, but which were excluded, and explain why they were excluded. | N / A |
| Study characteristics | 17 | Cite each included study and present its characteristics. | Table 1 |
| Risk of bias in studies | 18 | Present assessments of risk of bias for each included study. | Table 2 |
| Results of individual studies | 19 | For all outcomes, present, for each study: (a) summary statistics for each group (where appropriate) and (b) an effect estimate and its precision (e.g. confidence/credible interval), ideally using structured tables or plots. | Figures 2 - 6 |
| Results of syntheses | 20a | For each synthesis, briefly summarise the characteristics and risk of bias among contributing studies. | Table 1, Table 2, Figures 2 – 6, Supplementary Figures 1-25 |
|  | 20b | Present results of all statistical syntheses conducted. If meta-analysis was done, present for each the summary estimate and its precision (e.g. confidence/credible interval) and measures of statistical heterogeneity. If comparing groups, describe the direction of the effect. | P. 7 – 11, Figures 2 - 6, Supplementary Figures 1-25 |
|  | 20c | Present results of all investigations of possible causes of heterogeneity among study results. | Figure 3, P. 10 |
|  | 20d | Present results of all sensitivity analyses conducted to assess the robustness of the synthesized results. | Supplementary Figures 23 + 24 |
| Reporting biases | 21 | Present assessments of risk of bias due to missing results (arising from reporting biases) for each synthesis assessed. | N / A |
| Certainty of evidence | 22 | Present assessments of certainty (or confidence) in the body of evidence for each outcome assessed. | N / A |
| **DISCUSSION** | | |  |
| Discussion | 23a | Provide a general interpretation of the results in the context of other evidence. | P. 12 |
|  | 23b | Discuss any limitations of the evidence included in the review. | P. 13 |
|  | 23c | Discuss any limitations of the review processes used. | P. 13 |
|  | 23d | Discuss implications of the results for practice, policy, and future research. |  |
| **OTHER INFORMATION** | | |  |
| Registration and protocol | 24a | Provide registration information for the review, including register name and registration number, or state that the review was not registered. | P. 2 |
|  | 24b | Indicate where the review protocol can be accessed, or state that a protocol was not prepared. | P. 2 |
|  | 24c | Describe and explain any amendments to information provided at registration or in the protocol. | P. 2 |
| Support | 25 | Describe sources of financial or non-financial support for the review, and the role of the funders or sponsors in the review. | Funding section |
| Competing interests | 26 | Declare any competing interests of review authors. | P. 1 |
| Availability of data, code and other materials | 27 | Report which of the following are publicly available and where they can be found: template data collection forms; data extracted from included studies; data used for all analyses; analytic code; any other materials used in the review. | P. 1 |

*From:*  Page MJ, McKenzie JE, Bossuyt PM, Boutron I, Hoffmann TC, Mulrow CD, et al. The PRISMA 2020 statement: an updated guideline for reporting systematic reviews. BMJ 2021;372:n71. doi: 10.1136/bmj.n71 For more information, visit: <http://www.prisma-statement.org/>

**Supplementary table 2 – Search terms**

|  | Search terms |
| --- | --- |
| Population | (CAD OR CHD OR coronary OR cardiac OR heart OR ischemic OR CVD OR cardiovascular OR arteriosclerosis OR "arterial sclerosis" OR atherosclerosis) |
| Intervention | (wearable* OR "mhealth" OR smartwatch OR "wrist-worn" OR "fitness tracker" OR "fitness-tracker" OR "sports tracker" OR "sports-tracker" OR (Garmin AND (Vivofit OR Vivoactive OR Vivosmart OR Vivomove OR Vivoki)) OR "health monitor" OR "mobile monitor*" OR "mobile sens*" OR "biomedical sensor" OR "activity tracker" OR "activity monitor" OR "activity sensor" OR "remote monitor" OR "pedometer" OR "accelerometer" OR fitbit OR "miCoach" OR "apple watch" OR "Garmin" OR "Huawei" OR "Samsung gear" OR "polar watch" OR (polar AND (A300 or A360 or M200 or M400 or M430 or M430 or M460 or M600 or V650 or V800 or Loop2)) OR "smartcardia" OR "mi band" OR "miband" OR "Microsoft band" OR "Microsoftband" OR "Microsoft-band" OR "actiwatch" OR "Biobeat" OR "bioharness" OR "health watch" OR "Under armour" OR "tomtom" OR "withings" OR "amazfit" OR "physical activity tracker" OR "PA tracker") |
| Outcome | ("coronary event" OR "cardiovascular event" OR "cardiac event" OR "cardiac infarction" OR "myocardial infarction" OR MI OR "myocardial event" OR mortality OR death OR progression OR prognosis OR "hospitalization" OR "blood pressure" OR BMI OR "waist circumference" OR "walking distance" OR "cardiac fitness" OR VO2max OR HDL OR LDL OR "high-density lipoprotein" OR "low-density lipoprotein" OR cholesterol OR ApoB OR BNP OR "NT-proBNP" OR "B-type natriuretic peptide" OR "ankle-brachial pressure index" OR ABPI OR "ankle-brachial index" OR ABI OR microalbuminuria OR proteinuria OR revascularization) |
| Study type | ("randomized" OR "randomised" OR RCT) |
| Whole searchphrase | (CAD OR CHD OR coronary OR cardiac OR heart OR ischemic OR CVD OR cardiovascular OR arteriosclerosis OR "arterial sclerosis" OR atherosclerosis) AND (wearable* OR "mhealth" OR smartwatch OR "wrist-worn" OR "fitness tracker" OR "fitness-tracker" OR "sports tracker" OR "sports-tracker" OR (Garmin AND (Vivofit OR Vivoactive OR Vivosmart OR Vivomove OR Vivoki)) OR "health monitor" OR "mobile monitor*" OR "mobile sens*" OR "biomedical sensor" OR "activity tracker" OR "activity monitor" OR "activity sensor" OR "remote monitor" OR "pedometer" OR "accelerometer" OR fitbit OR "miCoach" OR "apple watch" OR "Garmin" OR "Huawei" OR "Samsung gear" OR "polar watch" OR (polar AND (A300 or A360 or M200 or M400 or M430 or M430 or M460 or M600 or V650 or V800 or Loop2)) OR "smartcardia" OR "mi band" OR "miband" OR "Microsoft band" OR "Microsoftband" OR "Microsoft-band" OR "actiwatch" OR "Biobeat" OR "bioharness" OR "health watch" OR "Under armour" OR "tomtom" OR "withings" OR "amazfit" OR "physical activity tracker" OR "PA tracker") AND ("coronary event" OR "cardiovascular event" OR "cardiac event" OR "cardiac infarction" OR "myocardial infarction" OR MI OR "myocardial event" OR mortality OR death OR progression OR prognosis OR "hospitalization" OR "blood pressure" OR BMI OR "waist circumference" OR "walking distance" OR "cardiac fitness" OR VO2max OR HDL OR LDL OR "high-density lipoprotein" OR "low-density lipoprotein" OR cholesterol OR ApoB OR BNP OR "NT-proBNP" OR "B-type natriuretic peptide" OR "ankle-brachial pressure index" OR ABPI OR "ankle-brachial index" OR ABI OR microalbuminuria OR proteinuria OR revascularization) AND ("randomized" OR "randomised" OR RCT) |

**Supplementary figure 1 – Meta-analysis of MVPA (min per week)**
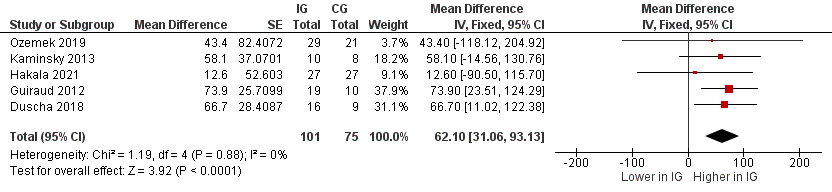


**Supplementary figure 2 – Meta-analysis of LPA (min per week)**


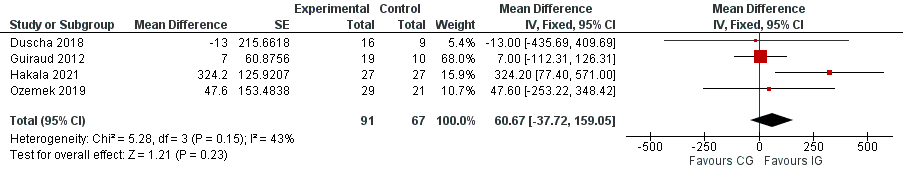


**Supplementary figure 3 – Meta-analysis of TPA (min per week)**
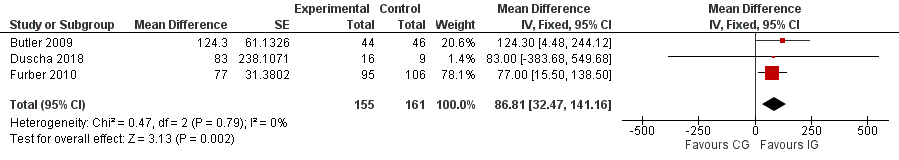


**Supplementary figure 4 – Meta-analysis of walking (min per week)**


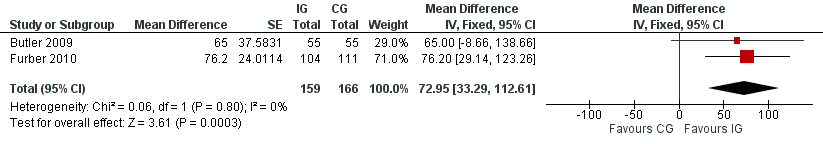


**Supplementary figure 5 – Meta-analysis of relative VO_2_peak [mL/kg/min]**


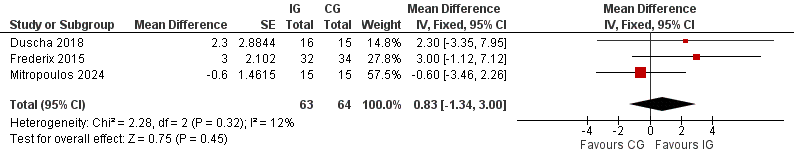


**Supplementary figure 6 – Meta-analysis of absolute VO_2_peak [L/min]**


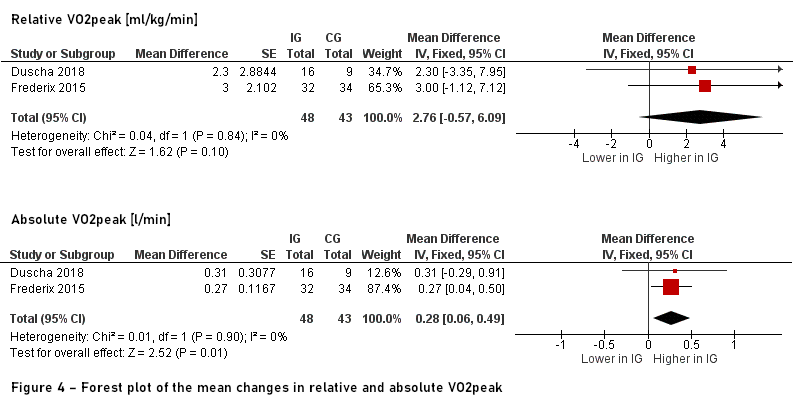


**Supplementary figure 7 – Meta-analysis of PPM by absolute VO_2_peak, METs at AT, MMP, and 6MWT**


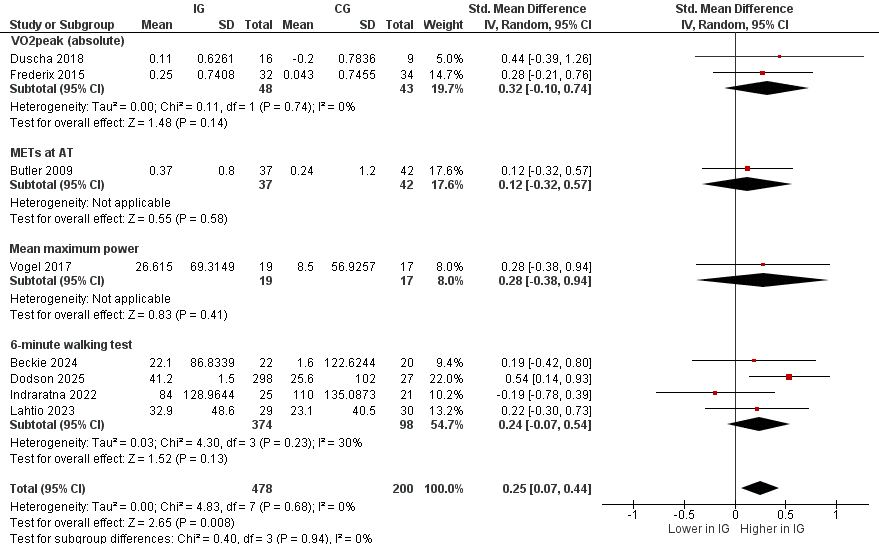


**Supplementary figure 8 – Meta-analysis of PPM by relative VO_2_peak, METs at AT, MMP, and 6MWT**


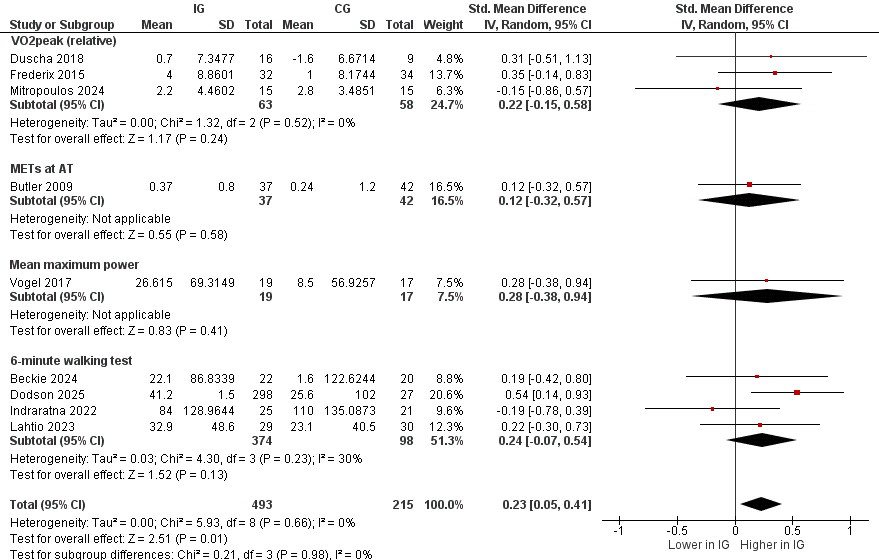


**Supplementary figure 9 - Meta-analysis of PPM by METs at AT, MMP, Peak RER, RERmax,** **and** **6MWT**


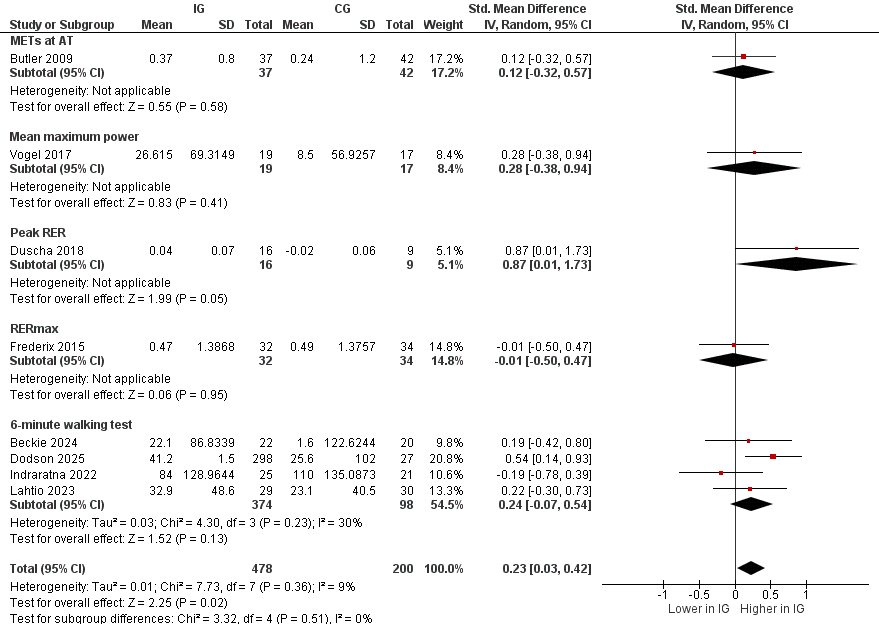


**Supplementary figure 10 - Meta-analysis of PPM by CPX, METs at AT, MMP, relative VO_2_peak, 6MWT**


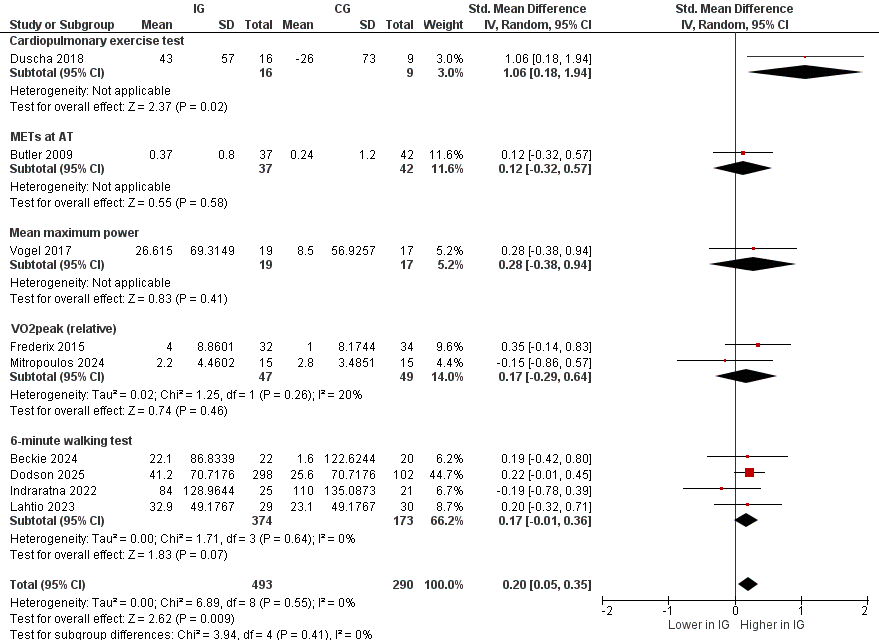


**Supplementary figure 11 – Meta-analysis of fatal cardiovascular events**


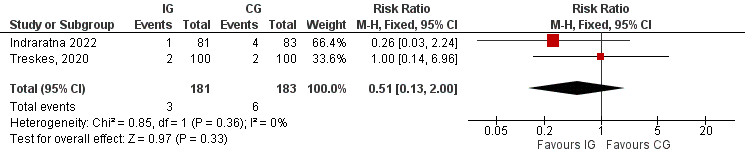


**Supplementary figure 12 – Meta-analysis of non-fatal cardiovascular events**


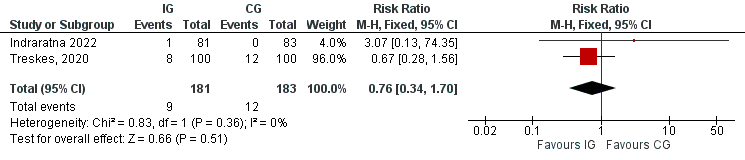


**Supplementary figure 13 – Meta-analysis of waist circumference (cm)**


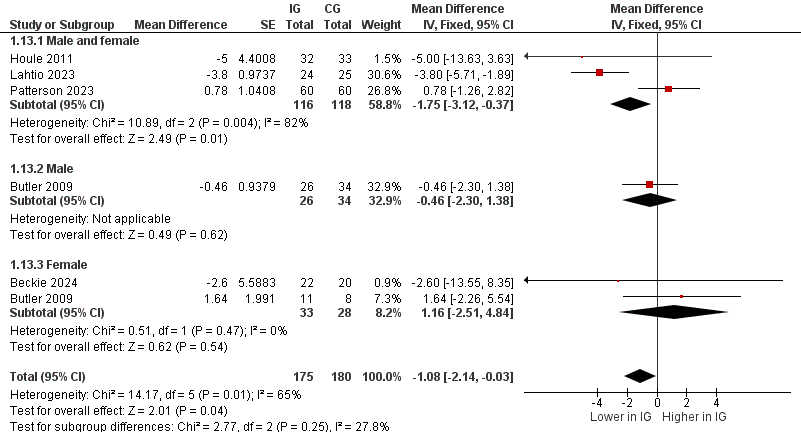


**Supplementary figure 14 – Meta-analysis of LDL (mg/dL)**

**
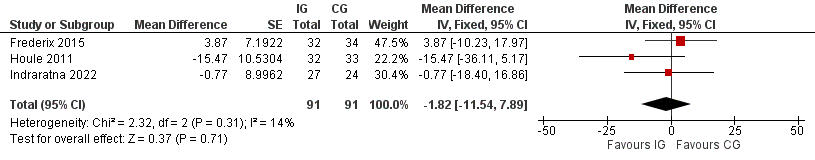
**

**Supplementary figure 15 – Meta-analysis of HDL (mg/dL)**

**
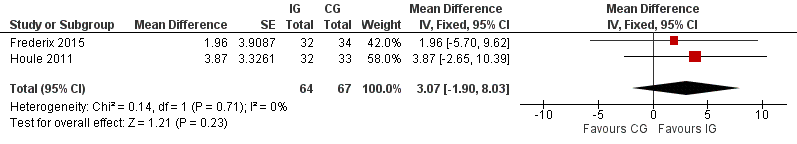
**

**Supplementary figure 16 – Meta-analysis of triglycerides (mg/dL)**

**
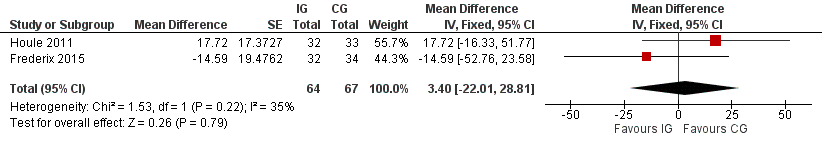
**

**Supplementary figure 17 - Meta-analysis of glucose (mg/dL)**

**
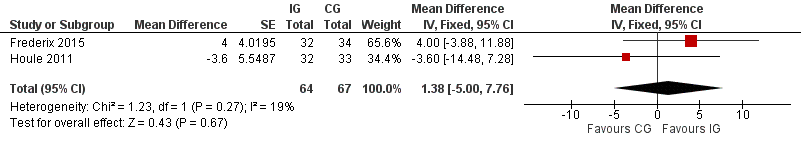
**

**Supplementary figure 18 - Meta-analysis of systolic blood pressure (mmHg)**


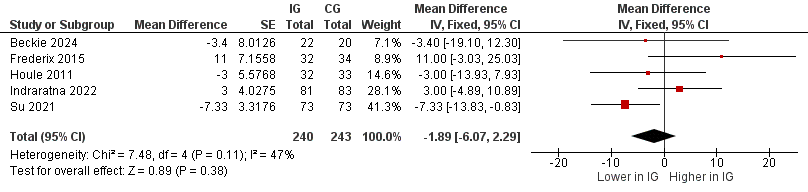


**Supplementary figure 19 - Meta-analysis of diastolic blood pressure (mmHg)**


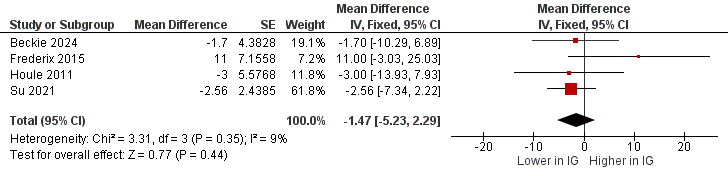


**Supplementary figure 20 – Meta analysis of sedentary time (min)**


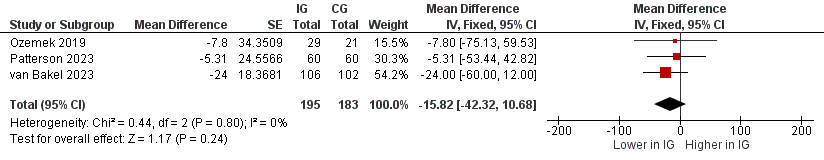


**Supplementary figure 21 – Meta analysis of BMI (kg/m²)**

**
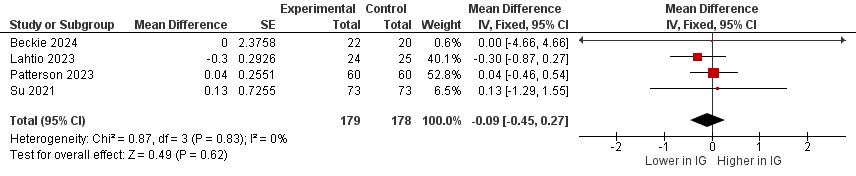
**

**Supplementary figure 22 – Meta analysis of 6 minute walking test (m)**


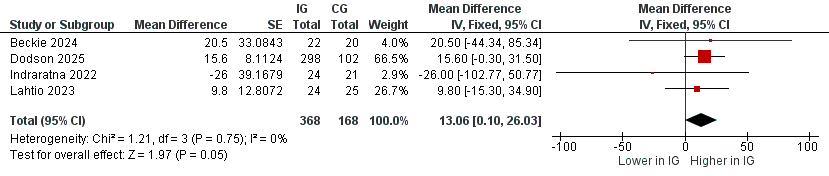


**Supplementary figure 23 - Sensitivity analysis of steps per day without outlier studies (in 1000 steps)**

**
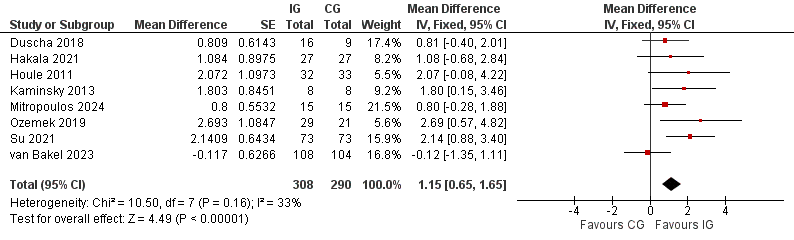
**

**Supplementary figure 24 – Sensitivity analysis of steps per day with random-effect model (in 1000 steps)**

**
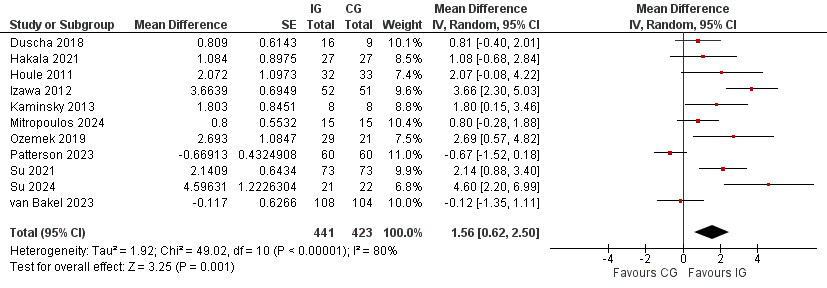
**

**Supplementary figure 25 – Subgroup analysis of steps per day by type of wearable (in 1000 steps)**


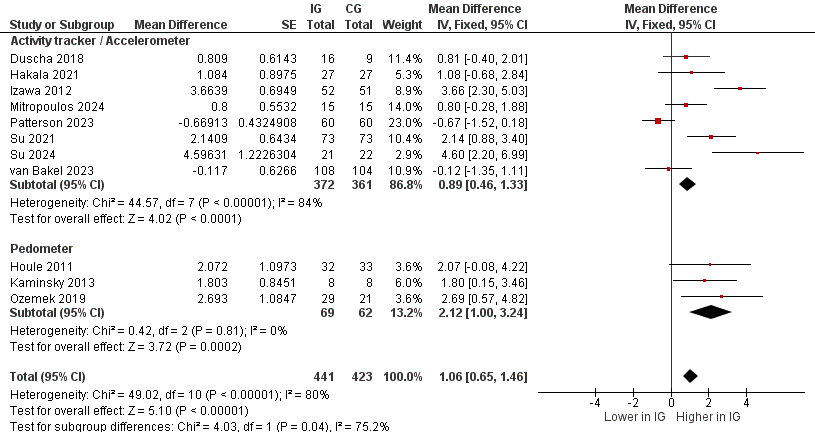


**Supplementary figure 26 – Funnel plot of included studies in meta-analysis of steps per day**


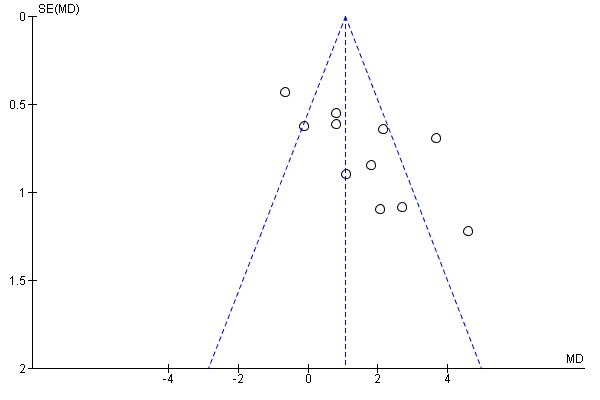

Supplement: Supplementary file 1 [file mmc1.docx]
